# Perinatal Mesenchymal Stromal Cells and Their Possible Contribution to Fetal-Maternal Tolerance

**DOI:** 10.3390/cells8111401

**Published:** 2019-11-07

**Authors:** Marta Magatti, Francesca Romana Stefani, Andrea Papait, Anna Cargnoni, Alice Masserdotti, Antonietta Rosa Silini, Ornella Parolini

**Affiliations:** 1 Centro di Ricerca E. Menni, Fondazione Poliambulanza Istituto Ospedaliero, 25124 Brescia, Italy; marta.magatti@poliambulanza.it (M.M.); francesca.stefani@poliambulanza.it (F.R.S.); andrea.papait@poliambulanza.it (A.P.); anna.cargnoni@poliambulanza.it (A.C.); antonietta.silini@poliambulanza.it (A.R.S.); 2 Istituto di Anatomia Umana e Biologia Cellulare, Università Cattolica del Sacro Cuore, 00168 Roma, Italy; alice.masserdotti@unicatt.it

**Keywords:** mesenchymal stromal cells, placenta, fetal-maternal tolerance, immunomodulation, pregnancy

## Abstract

During pregnancy, a successful coexistence between the mother and the semi-allogenic fetus occurs which requires a dynamic immune system to guarantee an efficient immune protection against possible infections and tolerance toward fetal antigens. The mechanism of fetal-maternal tolerance is still an open question. There is growing in vitro and in vivo evidence that mesenchymal stromal cells (MSC) which are present in perinatal tissues have a prominent role in generating a functional microenvironment critical to a successful pregnancy. This review highlights the immunomodulatory properties of perinatal MSC and their impact on the major immune cell subsets present in the uterus during pregnancy, such as natural killer cells, antigen-presenting cells (macrophages and dendritic cells), and T cells. Here, we discuss the current understanding and the possible contribution of perinatal MSC in the establishment of fetal-maternal tolerance, providing a new perspective on the physiology of gestation.

## 1. Immunology of Pregnancy

**Figure 1:**
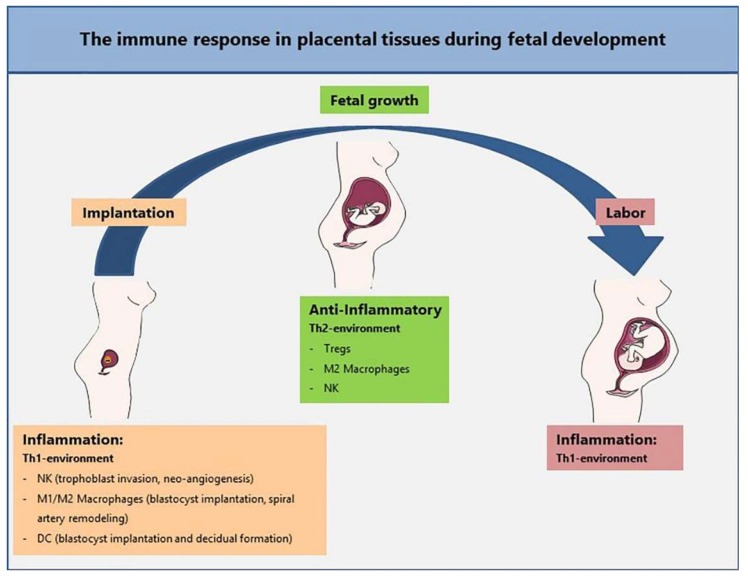
The immune response in placental tissues during fetal development. The first trimester of pregnancy is associated with a proinflammatory reaction which is necessary for implantation. In this phase, placental tissues are characterized by a T helper 1 (Th1) proinflammatory environment. Natural killer (NK) cells are involved in trophoblast invasion and support neoangiogenesis. A mixed M1 and M2 macrophage population mediates a delicate balance for blastocyst implantation and spiral artery remodeling. The second trimester is characterized by a Th2, anti-inflammatory microenvironment pivotal to fetal growth. T regulatory (Treg) cells, M2 macrophages, and NK cells support and promote the survival of the fetus. As gestation reaches the third trimester, an inflammatory and Th1-type immune state promotes the influx of immune cells into the myometrium which is crucial to foster labor and delivery.

In nature, pregnancy is a unique physiological phenomenon in which the mother coexists with the semi-allogeneic fetus that carries a combination of antigens half of which are paternal. For many years, Medawar’s paradox has provided the basis for the model that explains the cohabitation of mother and fetus [1]. Medawar proposed that the lack of fetal rejection by the mother might be explained by diverse mechanisms, which include: (a) the non-immunogenicity of the fetus due to antigenic immaturity, (b) the suppression of the mother’s immune system during pregnancy that in turn causes the mother’s immune system to “ignore” the fetus, (c) the uterus as an immunologically privileged site; and (d) the existence of an immunological barrier between mother and fetus, represented by the placenta [2]. Since the time of Medawar, the following has been demonstrated: (a) the fetus has immunogenic properties; (b) the maternal immune response is not suppressed during pregnancy; (c) the uterus does not represent an immune-privileged site and extrauterine pregnancies are also possible [3,4]; and (d) there are no physical barriers from immune cells in the placenta, and this is particularly true considering the human hemochorial placental arrangement [5], therefore, allowing bidirectional cell trafficking between the fetus and the mother [6]. Thus, placental tissue represents an active immunological site rather than a merely passive barrier from the mother. There is a great deal of evidence suggesting that a successful pregnancy requires a responsive and dynamic immune system from both the mother and the fetal and placental compartments in order to guarantee efficient immune protection against possible infections and tolerance toward fetal antigens. In line with the idea of a plastic immune remodeling, distinct immunological stages are identified during pregnancy, as previously extensively described [7,8] (Figure 1). At first, a local proinflammatory reaction accompanies the adhesion and invasion of the trophoblast to maternal tissues. Indeed, at the beginning of pregnancy, the maternal endometrium undergoes a process called decidualization, characterized by proliferation and differentiation of stromal cells. The stromal cells release cytokines and growth factors (mainly prolactin and insulin-like growth factor binding protein-1) that modify the extracellular matrix and expose adhesion molecules in order to create a receptive microenvironment for the attachment of the embryo, which occurs with the migration of fetal extravillous trophoblast cells to the decidua. Moreover, soon after implantation, the number of immune cells dramatically increases in the stroma of the decidua. In particular, the majority of first trimester human-decidual leukocytes are natural killer (NK) cells, followed by myeloid cells, T cells, and rare dendritic and B cells [9,10,11].

Between week 13 and week 27, the second trimester of pregnancy takes place. During this phase there is a shift from the initial proinflammatory to an anti-inflammatory and T helper 2 (Th2) response, aimed at preserving the fetal-placental system [12]. The tissue microenvironment is now enriched with M2-like macrophages and NK cells, whose interaction induces the generation of T regulatory (Treg) cells, key players in sustaining fetal-maternal tolerance [13,14].

Finally, in the third trimester, the return to an inflammatory and Th1-type immune state fosters labor and delivery. In this phase, infiltration of immune cells into the myometrium is crucial for promoting the contraction of the uterus, the delivery of the fetus, and the separation of the placenta. 

Throughout pregnancy, the immune system must engage in a fine-tuned balance and the immune cells, whether already present in placental tissue or recruited from peripheral blood, harbor peculiar features unique to their function and different from those from peripheral blood. 

The placental microenvironment is instrumental to the success of fetal-maternal tolerance and several mechanisms underlying immune regulation during pregnancy have been described, including the ability of perinatal mesenchymal stromal cells (MSC) to attract and educate leukocytes [8,15]. In this review, we discuss the involvement of stromal cells in the induction of fetal-maternal tolerance, with a focus on the crosstalk between MSC and immune cells.

## 2. Placental Structure and Perinatal MSC

**Figure 2:**
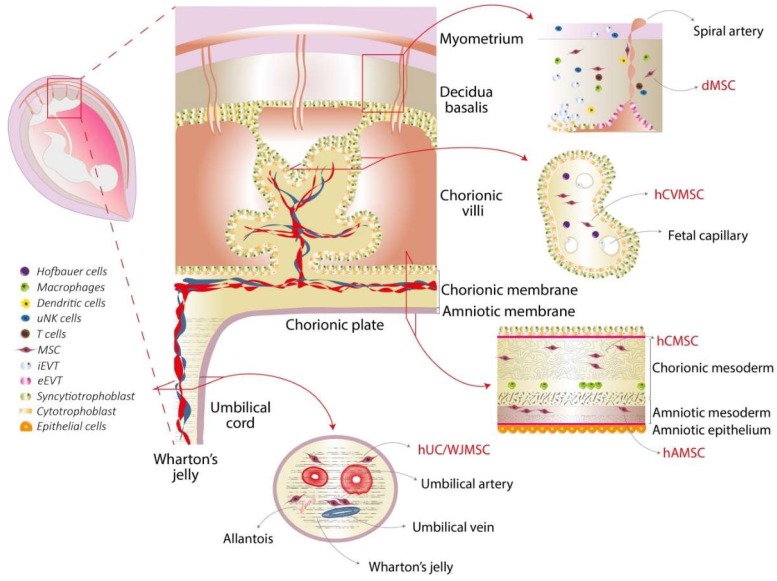
Placental structure and perinatal mesenchymal stromal cells (MSC). The placenta consists of complementary but distinct tissues, such as the decidua, the umbilical cord, the chorionic villi, and the chorionic and amniotic membranes. A schematic structure of each tissue is provided, and the immune cells and the MSC present in each tissue are indicated. Abbreviations: uterine natural killer (uNK) cells, invasive extravillous trophoblast (iEVT), endovascular extravillous trophoblast (eEVT), decidual MSC (dMSC), human chorionic villi MSC (hCVMSC), human chorionic MSC (hCMSC), human amniotic MSC (hAMSC), human umbilical cord/Wharton’s jelly MSC (hUC/WJMSC).

The placenta is a fetal-maternal organ that plays an essential role in fetal development, nutrition, and exchange of oxygen between the mother and the fetus. The fetal component of the placenta originates from the blastocyst, whereas the maternal component (decidua) is derived from the endometrium. Macroscopically, at term the placenta is discoid in shape with a diameter of 15 cm to 20 cm and a thickness of 2 cm to 3 cm. The surface that faces the fetus is called the chorionic plate, to which the umbilical cord is attached and the surface adjacent to the maternal endometrium is called the basal plate. Between these plates, there is a cavity, the intervillous space, which is located where maternal blood flows and is in contact with the chorionic villi that contain fetal blood [16,17] (Figure 2). 

The amniotic and chorionic fetal membranes enclose the fetus in the amniotic cavity. The amniotic membrane is composed of the following two main layers: the amniotic epithelium which is in direct contact with the amniotic fluid and the amniotic mesoderm which is comprised of collagens, fibronectin, and dispersed fibroblast-like cells, called amniotic MSC (hAMSC) [18]. The chorionic membrane features a mesodermal layer, adjacent but not fused to the amniotic stromal layer, which contains chorionic MSC (hCMSC) [18]. The distal part of the chorion (with respect to the fetus) is composed of a layer of extravillous, proliferating trophoblast cells interposed in varying amounts of Langhans’ fibrinoid, eventually covered by syncytiotrophoblasts.

Chorionic villi are finger-like structures that sprout from the chorion and anchor the placenta through the trophoblast of the basal plate to the maternal endometrium. Villi represent the functional units of placenta, where diffusion and active transport of nutrients and waste products takes place, and the maternal and fetal circulations are intimately juxtaposed. The core of the placental villi is enriched with MSC (termed chorionic villi MSC: hCVMSC) [19] and placental blood vessels, directly connected to the fetal circulation via the umbilical cord. The umbilical cord is inserted into the chorionic plate and contains one umbilical vein which conveys oxygenated and nutrient-rich blood from the chorionic plate to the fetus, and two umbilical arteries which transport de-oxygenated and nutrient-depleted blood back to the chorionic plate. The umbilical vessels participate in fetal-maternal circulation and are surrounded by a gelatinous, proteoglycan-rich matrix known as Wharton’s jelly. MSC can be isolated from both the entire umbilical cord and the Wharton’s jelly, termed umbilical cord (hUCMSC) and Wharton’s jelly MSC (hWJMSC), respectively [20,21]. 

The maternal component of the placenta, the decidua, is divided into three regions according to the relative position to the conceptus. The decidua basalis, which originated from the structural and functional transformation of the endometrium, represents the site where embryo implantation takes place and the basal plate is formed. The decidua capsularis grows over the embryo and encloses the chorion. The decidua parietalis lies on the opposite wall of the uterus and, by the fourth month of gestation, fuses with the decidua capsularis, thus, contributing to the disappearance of the uterine cavity. MSC have been isolated from both decidua basalis and parietalis (both referred to as dMSC) [22,23].

The MSC isolated from human placental tissues present phenotypes consistent with that of MSC from other human tissues [24]. To date, a consensus of the nomenclature of MSC from all the different perinatal tissues is missing, with the exception of cells isolated from the amniotic and chorionic membrane, which were described in the consensus from the *First International Workshop on Placenta-Derived Stem Cells* [18]. Therefore, the nomenclature used in this review was chosen on the basis of this consensus for hAMSC and hCMSC [18]. For the other MSC isolated from perinatal tissues, the following nomenclature was chosen: hUCMSC and hWJMSC for MSC from the umbilical cord and Wharton’s jelly, hCVMSC for MSC from the chorionic villi and the trophoblast compartment, and dMSC for MSC from the decidua. The origin (maternal or fetal), tissue of isolation (specific region of perinatal tissue), phenotype, and principal immunological functions reported for the perinatal MSC described in this review are summarized in Table 1. Generally, perinatal MSC are marked by the classical MSC proteins CD90, CD44, CD73, and CD105; lack hematopoietic markers CD45, CD34, CD14, and HLA-DR [18,19,25]; and harbor tri-lineage differentiation potential [18,25,26]. Nevertheless, discrete placental regions may give rise to MSC bearing distinct properties. For instance, hAMSC display attenuated growth kinetics and shorter lifespan as compared with hCMSC, dMSC, and hWJMSC [25,26], and exhibit lower osteogenic potential than WJMSC [27].

Of particular interest, perinatal MSC perform immunomodulatory functions through both cell–cell contact and paracrine signaling. Indeed, they carry out suppressive tasks, such as the inhibition of T and B cell proliferation, and boost the anti-inflammatory traits of monocytes, macrophages, dendritic cells, neutrophils, and NK cells. In addition, perinatal MSC induce Treg cells and anti-inflammatory M2 macrophages that are critical to maintain a balanced immune response [28,29]. The propensity of MSC in counteracting inflammation has fostered their implementation in several preclinical disease models where exacerbated inflammation is present, yielding, until now, promising results [29,30].

## 3. Role of MSC in Fetal-Maternal Tolerance

The critical core concept of a successful pregnancy is successful fetal-maternal tolerance. NK cells, antigen-presenting cells (APC) (macrophages and dendritic cells), and T cells patrol the fetal-maternal interface and actively sustain its homeostasis and development. Accordingly, the putative MSC-mediated immune regulation of pregnancy involves a concerted action with the major leukocyte subsets. The details of this framework and the differential contribution of the specific perinatal MSC subtype are discussed below.

### 3.1. Effect of Perinatal MSC on NK Cells

During the first trimester of pregnancy, the NK cells account for up to 70% of the lymphocytes present in the decidua, while, from the middle of the gestational period, they begin to gradually decrease, until becoming completely absent by the end of pregnancy [69,70]. They appear to play a fundamental role during pregnancy, including the production of chemokines and growth factors involved in trophoblast attraction and invasion, in the promotion of neo-angiogenesis and spiral artery remodeling [71], antimicrobial defenses, and the induction of tolerance at the maternal and fetal interface [72]. As matter of fact, alterations in decidual NK (dNK) cell numbers and activation status can play a role in pregnancy complications, such as immunologic infertility, recurrent spontaneous abortion, and preeclampsia [48,73]. In fact, pregnant mice that lack uterine NK cells present alterations in placental bed morphology and some of the pathological phenomena found in the preeclamptic uterus [74].

Human NK cells can be classified into two subsets according to CD56 (neural cell adhesion molecule 1, NCAM1) and CD16 expression. The majority of peripheral blood NK (pbNK) cells express low levels of CD56 and are positive for CD16 (CD56^dim^CD16^+^), as well as present high cytotoxic activity. NK cells express, acquire, and can adapt to various types of activating and inhibitory receptors which makes them able to address specific functions in different immunological settings. In fact, they can be sensors for microbial products and can be sentinels of virus infected cells, they can kill non-self and transformed cells which lack human leukocyte antigen (HLA)-I molecules and express ligands for activating NK receptors, and they can facilitate regulatory functions by producing cytokines [72,73,75,76,77]. 

Decidual NK cells are phenotypically different from the “classical” pbNK cells. Indeed, the majority of dNK cells are CD56^bright^CD16^−^. On the one hand, the dNK cells express a variety of activating receptors, including the entire natural cytotoxic receptor (NCR) repertoire (NKp30, NKp46, and NKp44), DNAM1, NG2C, NKG2D, and NKp80. On the other hand, they have a marked expression of inhibitory receptors, including the leukocyte immunoglobulin-like receptor subfamily B member 1 (LILRB1), which recognizes HLA-G, and KIR, in particular KIR2DL4, that binds to HLA-C molecules. Interestingly, both HLA-G and HLA-C are expressed by trophoblasts. Furthermore, although expressing high levels of cytotoxic perforin and cytolytic granules, dNK cells appear to be poorly cytotoxic, and produce low amounts of IFNγ as compared with pbNK cells. Therefore, the expression of inhibitory receptors, together with the poorly cytotoxic activity, could account for the preferential tolerance of dNK cells as compared with pbNK cells towards trophoblasts [78]. 

Different subsets of NK cells have been described in the decidua. Indeed, single-cell RNA sequencing of cells isolated from decidua and from the matched peripheral blood during the first trimester of pregnancy have demonstrated the existence of three different major NK cell subsets, such as dNK1, dNK2, and dNK3, bearing distinctive immunomodulatory profile [79]. In addition to NK cells, the human decidua also contains lymphoid tissue-inducer (LTi) like cells and innate lymphoid cells (ILC)3, the last of which includes both NCR^+^ and NCR^−^ cell subsets [80,81]. In particular, ILC3 are thought to play a relevant role in the induction and maintenance of pregnancy, partially mediated by the molecular interaction between PD-1 expressed on dNK and PD-1 ligand expressed by the invading trophoblast [82], moreover, pregnancy trained decidual NK (PTdNK) cells have been described [83]. Of note, PTdNK cells are present primarily in repeated pregnancies, and because repeated pregnancies are associated with improved placentation, this reinforces the notion of the critical role of dNK cells in proper placentation and ultimately in a successful pregnancy [83].

**Figure 3:**
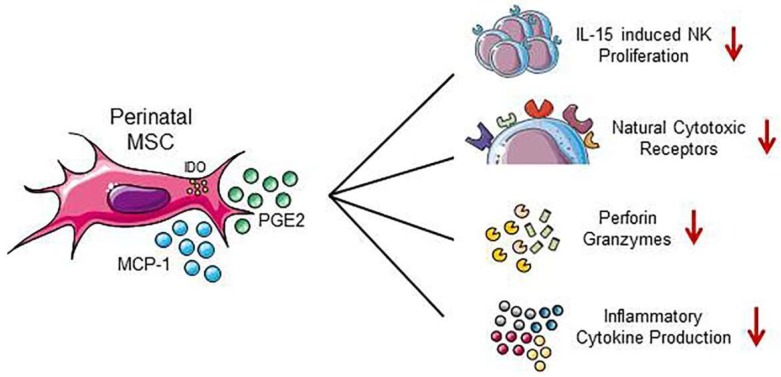
Effects of perinatal MSC on NK cells. The suppressive actions of MSC and their secreted factors on NK cell proliferation, phenotype, and activity are illustrated.

The interactions occurring between dNK cells and the surrounding microenvironment, including dMSC, appear to be the primary influence on the plasticity and the function of ILC, accounting for their heterogeneity [81] (Figure 3). Indeed, Vacca et al. showed that dMSC can sustain the differentiation of CD34^+^ cell precursors isolated from decidua towards functional (i.e., IL-8- and IL-22-producing) CD56^bright^CD16^−^KIR^+/−^ NK cells [32]. Moreover, the same group demonstrated that dMSC sharply suppress IL15-induced NK cell proliferation, inhibit the upregulation of activating receptors as well as the levels of perforin and granzymes, and reduce NK cell cytolytic activity and cytokine production [33]. Both indoleamine 2,3-dioxygenase (IDO) and prostaglandin E2 (PGE2), constitutively produced by dMSC, are involved in the dMSC-mediated inhibition of NK cell proliferation and cytotoxicity [33]. Furthermore, TGFβ released by dMSC has been reported to convert peripheral blood NK cells into decidual NK-like phenotype by triggering the expression of CD9 and KIR [84]. Moreover, dMSC are able to release a large number of soluble factors including prolactin, insulin-like growth factor binding protein 1 (IGF-1), VEGF, IL-11, and IL-15 that can improve lymphocyte viability and protect them from apoptosis [69]. Conditioned medium obtained from proliferative dMSC has been reported to inhibit the cytotoxicity of dNK cells [34], and MCP-1 released by decidual stromal cells has been shown to inhibit perforin expression in CD56^+^ NK cells, thus, reducing their cytolytic activity [35].

In addition to dMSC that are in close contact with dNK cells, hAMSC and hUCMSC/hWJMSC can also modulate NK cells by affecting their expansion and activity in vitro. As a matter of fact, hAMSC efficiently expand cord-blood NK progenitor cells in the presence of specific cytokines [55]. Moreover, both hAMSC and hUCMSC suppress the cytotoxicity and activation status of NK cells in a dose-dependent manner, mechanisms that correlate to the downregulation of CD69 and of the activating receptors NKp30, NKp44, NKp46, respectively [39,56]. In addition, hUCMSC and hAMSC significantly suppress the production of IFN-γ, TNF-α, and perforin in activated NK cells [40,41,42,56], and hUCMSC primed with IFN-γ display enhanced expression of HLA-ABC, thus, rendering them less susceptible to NK killing [41]. 

### 3.2. Effect of Perinatal MSC on Antigen-Presenting Cells (APC): Macrophages

APC represent an important component of the immunological milieu of pregnancy, providing a defensive immune response to pathogens and strongly contributing to fetal-maternal tolerance process [85]. Macrophages represent one of the most abundant APC, accounting for 20% to 25% of the total leukocyte population in the first trimester and maintaining their presence throughout pregnancy [9,86]. These cells are instrumental for successful implantation, placentation, fetal development, and parturition. In addition, they are involved in a multitude of activities, including extracellular matrix and spiral artery remodeling, clearance of apoptotic cells, tissue regeneration, fetal antigen recognition, inflammation, and immune modulation [87,88]. The crucial role played by macrophages during pregnancy is further supported by the fact that macrophage numbers are altered in different pregnancy-related complications, such as preeclampsia and chorioamnionitis [89,90], and aberrant macrophage activation is observed in preeclampsia, intrauterine growth restriction (IUGR), and preterm birth [91,92]. Because of their inherent plasticity, macrophages can acquire distinct cellular phenotypes, and thus promptly adapt and react to the surrounding environment. Macrophage functional spectrum ranges from proinflammatory M1 to anti-inflammatory tissue healing M2 cells, hence, describing the two major and opposing activities of these cells [93,94,95]. 

**Figure 4:**
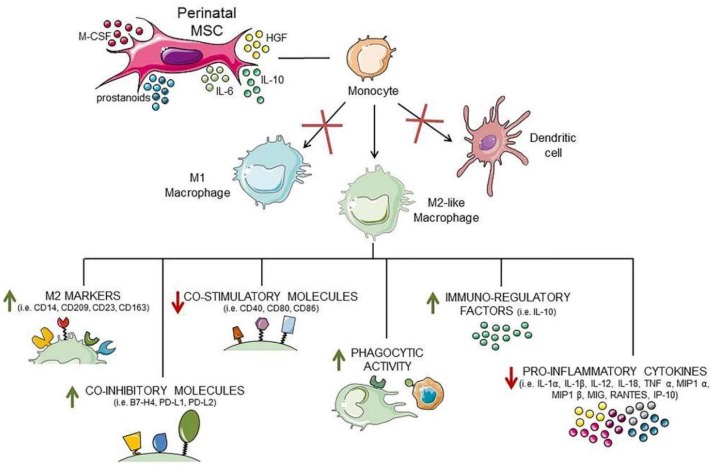
Effects of perinatal MSC on antigen-presenting cells. MSC and their secreted factors block dendritic cell and M1 macrophage differentiation, and induce the differentiation of monocytes into macrophages enriched with anti-inflammatory M2-like features. The suppressive or increased actions exerted by MSC on macrophages are represented.

In perinatal tissues, functionally different subsets of macrophages have been described [87,96]. During the peri-implantation period, decidual macrophages bear predominantly M1 traits. When extravillous trophoblast cells invade the uterine stroma decidual macrophages, a mixed M1 and M2 polarization pattern remains until mid-pregnancy, and, after the placental development is completed, decidual macrophages are predominantly skewed toward a M2 phenotype to prevent rejection of the fetus and ultimately impact parturition [97]. Macrophages are found in normal decidua [98,99], and decidual CD14^+^ monocytes and macrophages exhibit a signature typical of the M2 subtype, including the expression of CD206, CD209 [100], and the high production of the immunosuppressive cytokine IL-10 [101], confirming their important immunoregulatory role in the uterus during pregnancy. Despite the prevailing M2-like phenotype, early human decidual tissue has been shown to contain two distinct subsets of macrophages which are neither M1 nor M2 macrophages, indeed they both produce proinflammatory and anti-inflammatory cytokines and present similar phagocytic ability [88]. Similarly, Svensson et al. discriminated two decidual macrophage populations, one of which displayed a pronounced M2 phenotype [102]. The chorionic villi also contain macrophages, termed Hofbauer cells, endowed with micropinocytotic and phagocytotic ability, and likely implicated in angiogenesis and vasculogenesis of villi [103,104]. These cells possess a M2 phenotype [90,104]. Indeed, they are positive for CD209, CD163; negative for HLA-DR, -DP, and -DQ [105]; and produce PGE2 after in vitro stimulation with LPS [106]. Macrophages have also been described in the human mesodermal region within chorionic and amniotic membranes. These CD14^+^ macrophages, found to be of fetal origin [57], are positive for CD68, CD163, HLA-DR, and the co-stimulatory molecule CD86, whereas they lack CD80 and the dendritic cell markers CD1a and CD83 [57,107,108]. Since infection can be detrimental to pregnancy [109], it is also important that macrophages respond appropriately to pathogens or endogenous ligands. Indeed, evidence shows that macrophages from decidua [88], villi (Hofbauer cells) [90], or amniotic membrane [57] can be rapidly activated and respond to stimuli. The capacity of placental macrophages to acquire the unique phenotype described above, largely depends on the microenvironment created by the placenta, mainly via secreted factors [8] (Figure 4). Interestingly, when hCVMSC and hAMSC were added to monocytes differentiated in vitro into M1 macrophages, the monocytes underwent a phenotypical and functional switch toward macrophages with anti-inflammatory M2-like features. Indeed, monocytes exposed to hCVMSC or hAMSC expressed M2 markers similar to those expressed by macrophages found in placental tissues, such as CD14, CD209, CD23, and CD163, with an increased expression of co-inhibitory molecules B7-H4, PD-L1, PD-L2, and a decreased expression of co-stimulatory molecules CD40, CD80, and CD86. Moreover, monocytes differentiated in the presence of hCVMSC or hAMSC had higher phagocytic activity, produced higher IL-10 and lower proinflammatory cytokines. They were poor inducers of T cell proliferation and Th1 polarization, while able to increase the amount of Treg cell subset [49,51,67]. Moreover, even the phenotype of murine macrophages was shifted towards M2 when co-cultured with hUCMSC and hWJMSC [43] and hAMSC [65]. Importantly, hWJMSC and hUCMSC, hAMSC, and hCVMSC were able to shift both human monocyte-derived macrophages and murine macrophages towards M2 phenotype, also when cultured in the absence of cell–cell contact [43,50,67], or with the conditioned medium (CM) from unstimulated MSC [49,51,65,67], thus, supporting the fundamental role of constitutively-produced bioactive factors in the immunomodulatory activity of MSC [67]. Perinatal MSC express a broad spectrum of factors such as HGF, prostanoids (PGD2, PGF2a, and PGE2), IL-6, M-CSF, and IL-10 [53,58,63] that have been shown to modulate the immune functions of monocytes and macrophages [110,111] and enrich macrophages with M2-like phenotype and regulatory properties [102,112,113,114,115,116,117]. Moreover, prostaglandins, contained in CM from hAMSC, have been described to play an important role in directing the differentiation of distinct M2 subsets [51]. Furthermore, decidual stromal cells produced IL-34 able to polarize in vitro blood monocytes into macrophages bearing a phenotype (CD14^high^CD163^+^CD209^+^) and a cytokine secretion pattern similar to that of decidual macrophages [37].

The in vitro immunomodulatory actions of perinatal MSC on macrophages have been confirmed in in vivo studies, demonstrating the potential of perinatal MSC, or their CM, to educate macrophages to promote the resolution of injury. For example, hUCMSC which has been used to treat renal damage after ischemia and reperfusion were shown to reduce the infiltration of macrophages in injured tissues and increase the proportion of anti-inflammatory M2 macrophages, featuring suppression of IL-1β and IL-6 production and induction of IL-10 at the injury sites [43]. Furthermore, CM from hAMSC has improved wound closure in a mouse model of skin wound healing, dampening the M1 inflammatory response and inducing M2 macrophages [51]. Recently, human placenta-derived MSC-like cells have been reported to stimulate angiogenesis in a mouse model of hind limb ischemia and to lead to accumulation of M2-like macrophages in ischemic tissue [118]. Interestingly, hWJMSC have been found in the proximity of, or in contact with, lung and spleen macrophages upon intravenous administration in a chronic rat model of multiple sclerosis, corroborating the existence of crosstalk between MSC and monocytes/macrophages [44]. Consistently, macrophage depletion has impaired the therapeutic effects of hUCMSC in renal injury in vivo [43]. 

### 3.3. Effect of Perinatal MSC on APC: Dendritic Cells

Dendritic cells (DC) are second to macrophages among the APC, comprising 1.7% of the immune cells present during the first trimester of pregnancy [119]. DC have dichotomous functions. Immature DC capture antigens from invading pathogens or other foreign bodies and migrate to lymphoid tissues where they undergo complete maturation. This last process is associated with the loss of phagocytic receptors and upregulation of chemotactic signals (e.g., CCR7), T cell co-stimulatory molecules (CD80 and CD86), and activation molecules (CD83), along with changes in the HLA class II compartments. On the one hand, mature DC can prime effector T cell expansion and polarization towards Th1, Th2, or Th17 cells, as well as stimulate CD8^+^ T cytotoxic activities, and interact with B and NK cells. On the other hand, DC can promote immune tolerance by inducing effector T cell apoptosis and expansion of CD4^+^ Treg cells [120].

The role of DC in the placenta is less clear, although it has been demonstrated that uterine CD11c^+^ DC are crucial for implantation and early placentation, because the depletion of these cells in mouse models has led to failed implantation and embryo resorption [121]. Moreover, these cells can also be involved in remodeling of the maternal vasculature [121]. Furthermore, because uterine DC do not migrate to the lymph nodes, they can be trapped in the decidua, preventing the exposure of peripheral T cells to fetal antigens, and thus having an early role in promoting tolerance to paternal antigens [122,123]. Indeed, in the decidua of normal pregnancies, DC are locked in a tolerogenic state with an immature CD209^+^ and CD205^+^ phenotype unable to differentiate into mature CD83^+^ APC [124,125]. Although CD83^+^ proportions are similar between decidua and peripheral blood, decidual CD83^+^ cells have decreased secretion of IL-12p70 [70,124] and increased production of IL10 [126]. These results suggest that decidual DC cannot prime a fetal antigen–specific T cell response locally. Similarly to macrophages, the tolerogenic state of DC can be mediated by MSC present within the placenta. In fact, MSC from the umbilical cord, amniotic membrane, chorionic villi and decidua, and their conditioned medium, have been found to block the differentiation and maturation of monocytes towards DC in vitro (Figure 4). These cells did not express the DC marker CD1a, and the expression of the co-stimulatory molecules CD40, CD80, CD86, and CD83, as well as that of HLA-DR, was reduced [33,38,44,47,49,50,63,64,68,127]. In addition, the immunosuppressive traits, such as HLA-G or B7-H3 and B7-H4, involved in the protection of allogeneic transplants [128,129,130,131], were increased in the presence of hCVMSC [68]. Interestingly, PD-L2 (CD273) and PD-L1 (CD274) expression increased upon treatment with hCVMSC [68], and decreased with hAMSC (for PD-L1) [49] and hWJMSC (for PD-L2) [38], suggesting a different role for perinatal MSC in modulating myelocitic cell development.

Pregnancy-associated hormones, such as activin A, inhibin A, and the macrophage inhibitory cytokine-1 (MIC-1) expressed by trophoblast cells and decidual stromal cells can also affect the maturation and function of DC [132,133]. Here, the differentiation of DC was not simply blocked, but switched toward macrophages with anti-inflammatory M2-like features that expressed M2 markers CD14, CD163, and CD23, but lacked or expressed low levels of the M1 markers CD197 and CD80 [49,50], and had increased phagocytic activity [63]. They also produced lower levels of inflammatory cytokines IL-12p70, IL-23, TNF-α, CXCL10, MIG/CXCL9, CCL5, and MIP-1α, and higher levels of IL-6, MIC-1, MCP-1/CCL2, IL-1-β, IL-10, HLA-G, and PGE2 [50,63,64,68], and they expressed the immunosuppressive enzyme IDO [68]. Given these premises, we speculate that the differentiation of monocytes towards M2-like macrophages induced by MSC can account for the higher presence of M2 macrophages in pregnancy, as compared with DC. As a consequence, the myelocytic cells differentiated in the presence of MSC showed an impaired ability to stimulate allogeneic T cell proliferation [33,50,64,68,134], a characteristic similar to freshly isolated CD14^+^CD209^+^ decidual cells, described to efficiently take up antigens, but unable to stimulate naive allogeneic T cells [125]. It is also known that in response to trophoblast-derived supernatant, CD11c^+^ decidual DC are instructed to induce conversion of CD4^+^CD25^−^ T cells into CD4^+^CD25^+^FOXP3^+^ Treg cells [135]. 

Furthermore, the co-culture of T cells with DC in the presence of hCVMSC decreases the secretion of IL-12 and IFN-γ and induces high levels of IL-10 [68]. In addition, monocyte-derived DC, differentiated in the presence of MSC, acquired the ability to induce Th2 responses and Treg cells [134]. This induction of Treg cells resembles that obtained by the interaction between decidual NK and decidual CD14^+^ cells. Remarkably, other co-culture combinations involving either NK or CD14^+^ cells isolated from peripheral blood are ineffective to induce Treg cells, underlining the unique phenotypic and functional properties of decidual immune cells [136]. Monocytes are precursors of both DC and macrophages and this plasticity is conserved until the late stages of DC and macrophage differentiation [137,138]. Notably, the inhibitory effect of hAMSC on monocyte differentiation is not completely reversible [64] and, similarly, in the presence of DC-differentiating stimuli GM-CSF and IL-4, both CD14^+^ decidual and amniocorionic macrophages are unable to differentiate into DC [64,136]. 

### 3.4. Effect of Perinatal MSC on T Cells

T cells are moderately abundant in human decidua, accounting for 10% to 20% of the human decidua leukocyte population in the first trimester of pregnancy. As compared to peripheral blood, the decidua presents a different CD4:CD8 ratio and the frequency of the distinct subpopulation varies. Indeed, 30% to 45% of T lymphocytes is comprised of CD4^+^ cells and 45% to 75% is comprised of CD8^+^ cells. Within the CD4^+^ cell population, approximately 50% of the cells possess the activation and memory CD25^dim^ phenotype, 5% to 30% are Th1, 5% are Th2, and 2% are Th17 cells; within the CD8+ cells, 40% display the effector and memory CD28^−^ phenotype. A significant enrichment (5%) in CD25^bright^Foxp3^+^ Treg cells and a more homogenous suppressive phenotype was observed [13]. In addition, γδT cells, CD4−CD8−TCRαβ T cells, and NKT cells are rare in the decidua [13,139].

A full understanding of the physiological functions of the T cell repertoire has not yet been achieved, however, T cells may play a pivotal role in fulfilling a successful pregnancy [13]. The function of CD8^+^ effector T cells and Th17 cells is still unclear, although these cells may play a role in protecting the fetal-maternal interface from pathogens, thus, balancing fetal tolerance and antiviral immunity [140,141]. Several studies have shown that a proinflammatory Th1-dependent environment is required for the successful implantation of the blastocyst. Later on, development of the placenta is likely induced by progesterone and Th2-cytokines, with the induction of Treg cells, which likely exerts a prominent protective role when the maternal immune cells come into contact with fetal antigens, having the ability to suppress fetus-specific and nonspecific responses [14,85]. In fact, diminished decidua Treg cells during pregnancy complications such as preeclampsia and after recurrent spontaneous miscarriages have been observed [142,143]. Once again, the microenvironment created by perinatal MSC seemed to dictate the recruitment, expansion, and phenotype of T cells. For example, silencing dMSC gene expression of chemokines (e.g., CXCL9, CXCL10, and CXCL11) limited T cell access to the fetal-maternal interface in mice [144]. 

**Figure 5:**
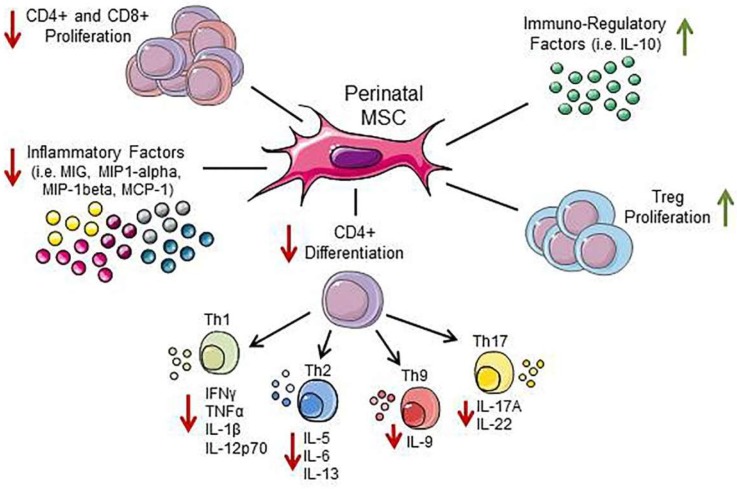
Effects of perinatal MSC on T cells. MSC and their secreted factors suppress the proliferation, inflammatory cytokine production, and differentiation of T cells, while they stimulate the generation of Treg cells and factors.

Several in vitro studies have focused on the impact of different perinatal MSC populations on T lymphocyte functions [28] (Figure 5). Specifically, perinatal MSC and their CM, strongly suppress T lymphocyte proliferation induced in vitro by a variety of stimuli (allogeneic stimulus, T cell receptor cross-linking, mitogens, and recall antigen) [31,36,52,53,54,57,58,59,60,66,127]. Both fetal- and maternal-derived MSC (hAMSC and dMSC, respectively) showed a dose-dependent inhibition of the proliferation of T cells activated by an allogeneic stimulus (mixed lymphocyte reaction, MLR). Nevertheless, fetal MSC demonstrated a significantly higher inhibitory capacity as compared with maternal MSC, and this stronger inhibition by fetal MSC was even more prominent in a secondary MLR with primed alloreactive T cells [31], consistent with a protective mechanism adopted by fetal MSC towards the mother’s activity. In this context, placenta-derived MSC, including hAMSC and their CM, were able to effectively inhibit Th1 and Th17 differentiation [49,62,127], while having no effect on the Th2 population [49,62]. In addition, a wide range of T cell subset related cytokines, such as Th1 (IFNγ, TNFα, IL-1β, and IL-12p70), Th2 (IL-5, IL-6, and IL-13), Th9 (IL-9), and Th17 (IL-17A and IL-22) cytokines were inhibited [45,49,52,61,62,127], as well as other inflammatory factors [60]. It is worth noting that although hAMSC and their CM decrease IL-17 secretion [45,52,61,62], hWJMSC and hCVMSC induce the production of IL-17 in MLR and promote the release of low levels of IL-17 from unstimulated PBMC [45]. On the one hand, the function of Th17 in pregnancy is unclear, and whether IL-17 and Th17 suppression or promotion is involved in the immunosuppressive capacity of MSC requires further investigation. On the other hand, hAMSC and their CM possess the ability to promote the development of Treg cells, and to stimulate the secretion of IL-10 [49,61,62,145]. It is believed that the decidual enrichment of Treg cells is due to the local proliferation of these cells, as judged by their frequent expression of Ki-67 (a marker for cell proliferation) [13], and therefore how perinatal MSC contribute to this proliferation needs to be determined.

Consistent with in vitro studies, hAMSC treatment significantly decreased the production of Th1 (IFN-γ) and Th17 (IL-17) cytokines and increased the levels of IL-10 in a mouse model of collagen-induced arthritis. Furthermore, they induced the generation of antigen-specific Treg cells in the periphery that were able to prevent arthritis progression [61]. In a rat model of multiple sclerosis, the therapeutic potential of hWJMSC correlated to their ability to modulate T cell (splenocyte) proliferation [44]. In addition, hyperglycemic improvement, related to the immunomodulatory effects exerted by hWJMSC, has also been reported after cell transplantation into diabetic animal models [46,48]. Indeed, the authors demonstrated reduced systemic and pancreatic levels of specific T subsets, such as Th1 and Th17, involved in the pathogenesis of type I diabetes in NOD mice. Finally, a shift toward the Th2 profile and an increased proportion of Treg cells were also found in hWJMSC-treated mice [48].

## 4. Conclusions

In this review, we highlight the significant contribution of perinatal MSC in shaping the placental microenvironment, and thus triggering the unique phenotype and properties of myeloid and lymphoid cells that sustain fetal-maternal tolerance. In vitro and in vivo studies have demonstrated that MSC can impact NK cell functions, guide macrophage and dendritic cell commitment, as well as govern T cell proliferation and subtype specification in the placental tissue. As a matter of fact, immune cell depletion led to failed implantation and embryo resorption (in mouse models) [121], altering immune cell numbers and the activation status which entailed diverse human pregnancy complications, such as preeclampsia, chorioamnionitis, intrauterine growth restriction, and preterm birth [89,91,92,146,147].

The specific role of perinatal MSC in pregnancy seems to be partly tissue specific, as cells that originate from different perinatal sources exhibit distinct behaviours. For instance, hWJMSC and hCVMSC promote IL-17/Th17 on stimulated PBMC [45] while hAMSC mediate their suppression [49,62]; hAMSC, hCVMSC, and hWJMSC differentially modulate the expression of PD-L2 and PD-L1 on dendritic cells [38,49,68]; and fetal MSC display a higher capacity to inhibit T cell proliferation as compared to maternal MSC [31].

Several mechanisms involved in the immune regulation during pregnancy are described, which include epigenetic modifications [148], microRNAs [149], histocompatibility antigens [150], and the action of microbiota [7]. Indeed, prenatal exposure to an agriculture environment (bacteria, pesticides, etc.), or maternal diet, affects the epigenetic signatures at the important immune loci in placenta [151,152], or cord blood [153], and changes in the epigenetics of perinatal tissues have been shown to be associated with disease susceptibility in childhood [154]. Similarly, exposure to the maternal microbiota during pregnancy can markedly affect the early development of the postnatal immune system in the offspring [155]. How these mechanisms interact and consequently affect the immunomodulatory properties of perinatal MSC remain a matter for study. For example, epigenetic silencing of key chemokine genes in mice has been shown to reduce the ability of dMSC to support the recruitment of effector T cells within the decidua, thus, supporting fetal immune tolerance [144]. Whether these epigenetic modifications also occur in human decidua is still unknown. Moreover, altered miRNome [156], proteome [157], and bioenergetic profiles [158] in UCMSC and hAMSC were shown to be associated with maternal obesity, and these alterations were suggestive of a defective response to oxidative stress in these cells [158], whereas their contribution in the immunomodulatory properties of perinatal MSC is not clearly defined. Inquiring into the immunomodulatory properties of perinatal MSC from normal and complicated pregnancies could help dissect their role in disease and, ultimately, in fetal-maternal tolerance. In addition, the epigenetic state of fetal genes has been shown to be altered during gestational diabetes mellitus (GDM) [159], and CM from hAMSC and hCMSC from healthy and GDM pregnancies show an equivalent immunoregulatory effect on modulating T cell (Jurkat) proliferation and cytokine secretion [160]. Similarly, both hAMSC and hCMSC from normal and GDM pregnancies have the ability to affect macrophage cytokine secretion, but hCMSC from GDM pregnancies had a reduced effect on macrophage regulation as compared with those from normal pregnancies [160]. Likewise, hAMSC from preeclamptic placenta feature similar immune modulatory [49] and cytokine profile [161] to cells from normal pregnancies, thus, suggesting a potential for these cells in efficiently counteracting the inflammatory environment and, ultimately, contributing to fetal survival. Conversely, dMSC derived from pregnancies with preeclampsia have significantly reduced levels of sICAM and SDF-1 as compared with those from normal pregnancies [161], and thus likely differentially contributing to the immunological framework. 

Given that MSC from decidua and trophoblast tissues stand at the fetal-maternal interface, where tolerance events may occur in the placenta, it is reasonable to assume that they are more intimately involved in this process [162,163]. However, the fact that MSC, derived from both the amniotic membrane and the umbilical cord, present high immunosuppressive and regulatory features supports the hypothesis that all the perinatal MSC, also those located internally to the trophoblast and the maternal decidua, may be implicated in this critical activity. Acquiring the exact position of perinatal MSC within the complex functional and dysfunctional networks during pregnancy merits further investigation and could help in comprehending the fascinating phenomenon of fetal-maternal tolerance, offering new information for the design of novel therapeutic approaches for pregnancy complications.

## Figures and Tables

**Table 1:** Perinatal mesenchymal stromal cells (MSC).

Origin	Tissue	Acronym	Phenotype	Function
Maternal	Decidua	dMSC	Positive for: CD44 [22,23,25,31], CD29 [25,31], CD54 [31], CD73 [25,31], CD90 [22,23,25,31], CD105 [22,23,25,31], CD146 [22,23], CD166 [22,23,31], HLA-A,B,C [22,23,31] Negative for: CD3 [25], CD11b [25], CD14 [22,23,25,31], CD19 [22,23,25], CD25 [31], CD31 [22,23,31], CD34 [25,31], CD40 [22,23,31], CD45 [22,23,25,31], CD56 [25], CD80 [22,23,31], CD83 [22,23], CD86 [22,23,31], CD117 [31], CD123 [31], CD133 [31], HLA-DR [22,23,25,31], HLA-G [25,31]	Differentiation of CD34+ precursor cells into functional natural killer (NK) cells [32]
				Inhibition of NK cell proliferation and cytotoxicity [33,34,35]
				Inhibition of T cell proliferation [31,33,36]
				Inhibition of dendritic cell (DC) differentiation [31,33]
				Induction of M2 macrophage phenotype [37]
Fetal	Umbilical cord	hUCMSC	Positive for: CD73 [25,26], CD90 [25,26], CD105 [25,26], CD29 [25], CD44 [25], CD49d [26], CD56low [25], CD133 [21], CD142 [26], CD146 [26], CD166 [26], CD200 [26], CD235a [21], CD349 [26], B7-H1/PD-L1 [38], HER1 [26], HER2 [26], MCSP [26], cMet [26] Negative for: CD3 [25], CD11b [25], CD14 [25,26], CD19 [25,26], CD31 [26], CD34 [25,26], CD45 [25,26], CD80 [38], CD86 [38], CD136 [26], CD143 [26], CD271 [26], HLA-DR [25,26,38], HLA-G [25], B7-DC/PD-L2 [38]	Inhibition of NK cell cytotoxicity and activation [39,40,41,42]
				Expansion of T regulatory (Treg) cells [38]
				Inhibition of monocyte-derived DC differentiation [38]
				In a murine model of renal damage, decrease of macrophage infiltration [43]
				Induction of M2 macrophages [43]
	Wharton’s jelly	hWJMSC	Positive for: CD10 [20,21], CD13 [20,21], CD29 [20,21], CD44 [20,21], CD49e [20], CD51 [20,21], CD68 [20,21], CD73 [20,21], CD80 [20], CD90 [20,21], CD105 [20,21], CD117 [20], CD166 [20,21], CD200 [44], ALP21, CK8 [20], CK18 [20], CK19 [20], Connexin-43 [20], COX-2 [44], DNMT3B [21], GABRB3 [21], GATA-4 [20], GATA-5 [20], GATA-6 [20], GFAP [20], HLA-A,B,C [20,21], HLA-E [44], HLA-G [20,21,44], HNF-4α [20], IDO-1 [44], Nanog [20,21], Nestin [20], NSE [20], OCT3/4A [20], CD274/PD-L1 [44], REX2 [21], α-SMA [20], SOX2 [21], SSEA-4 [21], Tra-1-60 [21], Tra-1-81 [21], TSG-6 [44], Vimentin [20] Negative for: CD14 [20,21], CD31 [20,21], CD33 [20], CD34 [20,21], CD45 [20,21], CD56 [20,21], CD86 [20], CD163 [20], CK-7 [20] Discordant markers: HLA-DR (Negative [20] or positive [21])	Inhibition of T cell proliferation [36,44,45,46]
				Inhibition of monocyte-derived DC differentiation [44,47]
				In diabetic NOD mice, reduction of systemic and pancreatic levels of T helper 1 (Th1) and Th17, shift toward Th2, increment of Treg cell levels, decrease of DC [48]
	Amnion	hAMSC	Positive for: CD10 [18,26,49,50], CD13 [18,27,49,50,51], CD26 [49], CD29 [18,25,27,31,52], CD44 [18,25,27,31,49,50,52], CD49a [49,50], CD49b [49,50], CD49c [18,49,50], CD49d [18,26,49,50], CD49e [18,50], CD49f [50], CD54 [18,31], CD59 [49], CD73 [18,25,26,27,31,36,49,50,51,52,53,54], CD85a [49], CD90 [18,25,26,27,31,36,49,50,51,52,53,54], CD105 [18,25,26,27,31,36,49,50,52,53,54], CD140b [18,49], CD142 [26], CD146 [26,49], CD166 [18,26,27], CD200 [26,49], CD252 [49], CD272 [49], CD273/PD-L2 [49], CD274/PD-L1 [49], CD284 [49], CD349 [18,26], HER1 [26], HER2 [26], HLA-A,B,C [18,27,49,50,52,54], MCSP [26], cMet [26], Oct-4 [18] Negative for: B7-H4 [49], CD3 [18,25], CD11b [25], CD14 [18,25,26,31,50,53,54], CD19 [25,26,53], CD25 [31], CD31 [18,26,31,49,52], CD33 [49], CD34 [18,25,26,31,36,49,52,53,54], CD39 [49], CD40 [31], CD45 [18,25,26,31,36,49,50,52,53,54], CD52 [49], CD70 [49], CD80 [27,31], CD86 [27,31], CD106 [49], CD109 [49], CD114 [49], CD116 [49], CD117 [31,49,50], CD120b [49], CD123 [31], CD124 [49], CD133 [18,31], CD136 [26], CD143 [26], CD146 [50], CD152 [49], CD154 [49], CD244 [49], CD271 [49], CD275 [49], CD282 [49], CD324 [18], CD326 [50], HLA-DM [49], HLA-DQ [49], HLA-DR [18,25,26,27,31,50,52,53,54], HLA-G [25,31,49] Discordant markers: CD271 (low [18] or negative [26]), CD166 (weakly positive [31,50] or positive [49])	Expansion of cord-blood NK cells in the presence of specific cytokines [55]
				Inhibition of NK cell cytotoxicity and activation [56]
				Inhibition of T cell proliferation [31,36,49,52,53,54,57,58,59,60]
				Expansion of Treg cells [49,61]
				Inhibition of Th1, Th17 formation [49,61,62]
				Inhibition of inflammatory cytokines [45,49,52,60,61,62]
				Inhibition of monocyte-derived DC differentiation [49,50,63,64]
				Inhibition of monocyte-derived M1 differentiation [49,51]
				Induction of M2 macrophage phenotype [49,50,51,65]
	Chorion	hCMSC	Positive for: CD10 [18], CD13 [18], CD29 [18,25,66], CD44 [18,25,66], CD49e [18], CD54 [66], CD73 [18,25,53,66], CD90 [18,25,53,66], CD105 [18,25,53,66], CD140b [18], CD166 [18], CD349 [18], CD271low [18], HLA-A,B,C [18] Negative for: CD3 [25,66], CD11b [25], CD14 [18,25,53,66], CD19 [25,53], CD34 [18,25,53,66], CD45 [18,25,53,66], CD56, [25], CD117 [18], CD133 [18], CD324 [18], HLA-DR [18,25,53], HLA-G [18,25]	Inhibition of T cell proliferation [53,66]
	Chorionic villi	hCVMSC	Positive for: CD29 [45], CD44 [45,67,68], CD73 [45], CD90 [45,67,68], CD146 [67,68], CD166 [67,68], CD105 [45,67,68], HLA-class I [45] Negative for: CD14 [45], CD19 [67,68], CD31 [45], CD34 [45], CD40 [67,68], CD45 [45,67,68], CD80 [67,68], CD86 [67,68], CXCR4 [45], HLA-DR [45,67,68], SSEA-3 [45], SSEA-4 [45]	Inhibition of monocyte-derived DC differentiation [68]
				Inhibition of monocyte-derived M1 differentiation [67]
				Induction of M2 macrophage phenotype [67]
				Decreased secretion of IL-12 and IFN-gamma when co-cultured with T cell and DC [68]
				Promotion of IL-17/Th17 [45]
